# Supplementary material for: Maternal perception, barriers, and facilitators regarding oral rehydration salt solution in diarrhoeal disease: A qualitative study in Bangladesh
Source: PLoS One. 2025 Jun 3;20(6):e0325386. doi: 10.1371/journal.pone.0325386 (PMC12132974; doi:10.1371/journal.pone.0325386)
Supplement: S2 File — (PDF) [file pone.0325386.s002.pdf]

Protocol No. PR-23138	Version No. 3.0	Date: 06/01/2024

Protocol Title: Assessment of maternal knowledge, attitude, and practice regarding commercially available oral rehydration solution preparation and its use among under-5 children suffering from diarrhoeal illnesses at Dhaka Hospital, icddr,b.

In-depth interview guidelines

Information of the participant

অংশগ্রহণকারীর তথ্য

Study ID:
Name
নাম
Age
বয়স
Sex of the child
শিশুর লিঙ্গ
Age of the child
শিশুর বয়স
Educational qualification of the mother
মায়ের শিক্ষাগত যোগ্যতা
Educational qualification of the father
বাবার শিক্ষাগত যোগ্যতা
Occupation of the mother
মায়ের পেশা
Occupation of the father
বাবার পেশা

Information regarding knowledge, attitude and practice about ORS preparation and use

মুখে খাওয়ার স্যালাইন এবং তার প্রস্তুতপ্রণালী সম্পর্কিত জ্ঞানের ব্যাপারে তথ্যাবলী

Q1. Please tell us about the problems regarding which you got admitted to this hospital.

প্রশ্ন ১: আপনি বর্তমানে যে সমস্যা নিয়ে এই হাসপাতালে ভর্তি হয়েছেন সে সম্পর্কে আমাদের কিছু বলুন।

Q2. Have you heard about ORS or Oral saline? Please tell us in details about what you know about it?

প্রশ্ন ২: আপনি কি ওর-স্যালাইন বা মুখে খাবার স্যালাইনের নাম শুনেছেন? এটি সম্পর্কে যা জানেন দয়া করে বিস্তারিত বলুন।

[Please probe where necessary]

[প্রয়োজনে অতিরিক্ত প্রশ্ন করুন]

তথ্যের উৎস:

কখন ওআরএস ব্যবহার করতে হয়:

ওআরএসের উপকারীতা:

অন্যান্য:

Q3. Please tell us about the advantages or disadvantages of using ORS?

প্রশ্ন ৩: খাবার স্যালাইন ব্যাবহারের উপকারিতা বা অপকারিতা সম্পর্কে দয়া করে আমাদের কিছু বলুন।

[Please probe where necessary]

[প্রয়োজনে অতিরিক্ত প্রশ্ন করুন]

খরচ প্রসঙ্গে:

বানানো এবং ব্যাবহারের সুবিধা প্রসঙ্গে:

সংরক্ষণের সুবিধা প্রসঙ্গে:

অন্যান্য:

Q4. Please tell us about how to prepare packet oral saline that is available in shop/pharmacy?

প্রশ্ন ৪: দোকান বা ফার্মেসিতে যে স্যালাইন (প্যাকেটজাত) পাওয়া যায়, সেটা কীভাবে প্রস্তুত করতে হয় সে সম্পর্কে দয়া করে আমাদের বলুন।

[Please probe where necessary]

[প্রয়োজনে অতিরিক্ত প্রশ্ন করুন]

স্যালাইন প্রস্তুত করার পূর্বে (সাবান-পানি দিয়ে হাত ধোয়া):

প্রস্তুত করার পাত্র সম্পর্কে (সাবান-পানি দিয়ে পাত্র ধোয়া):

প্রস্তুত করার পানি সম্পর্কে (সাপ্লাই পানি, টিউবওয়েল, ফোটানো পানি, ফোটানোর পর ঠান্ডা করা পানি, বোতলজাত পানি):

প্যাকেটে লেখা প্রণালী অনুসরণ সম্পর্কে (তারিখসহ):

প্যাকেটের সবটুকু পাউডার ব্যবহার সম্পর্কে (এবং ভালোভাবে না গোলা পর্যন্ত নাড়ানো সম্পর্কে):

অন্যান্য:

Q5. Please tell us about how to prepare ORS using ingredients found at home?

প্রশ্ন ৫: বাসায় পাওয়া উপাদান দিয়ে স্যালাইন কীভাবে প্রস্তুত করতে হয় সে ব্যাপারে দয়া করে আমাদের বলুন।

[Please probe where necessary]

[প্রয়োজনে অতিরিক্ত প্রশ্ন করুন]

উপকরণগুলোর নাম (পানি—কোন ধরনের, ঘরে বানাবার উপকরণ):

উপকরণগুলোর পরিমাণ:

প্রস্তুতের প্রণালী:

অন্যান্য:

Q6. Please tell us about the process of giving ORS to a sick child?

প্রশ্ন ৬: অসুস্থ শিশুকে স্যালাইন দেবার পদ্ধতি সম্পর্কে দয়া করে আমাদের কিছু বলুন।

[Please probe where necessary]

[প্রয়োজনে অতিরিক্ত প্রশ্ন করুন]

কতটুকু দিতে হয়:

কীভাবে দিতে হয়:

কখন দিতে হয় (প্রতিবার পায়খানার পরে কি না):

ডায়রিয়া না থাকলেও স্যালাইন দেওয়া যায় কি না:

কতদিন দিতে হয়:

বাচ্চা আরও খেতে চাইলে কি আপনি তাকে স্যালাইন প্রদান করবেন?:

কোন ধরনের পাত্র ব্যবহার করতে হয়:

অন্যান্য:

Q7. Please tell us how to store ORS?

প্রশ্ন ৭: খাবার স্যালাইন কীভাবে সংরক্ষণ করতে হয় সে সম্পর্কে আমাদের কিছু বলুন।

[Please probe where necessary]

[প্রয়োজনে অতিরিক্ত প্রশ্ন করুন]

পাত্রের ধরন:

সময়:

অন্যান্য:

Q8. What is your opinion on Commercial and Home-made ORS?

প্রশ্ন ৮: দোকান থেকে কেনা স্যালাইন এবং বাসায় তৈরি স্যালাইন সম্পর্কে আপনার অভিমত ব্যক্ত করুন।

[Please probe where necessary]

[প্রয়োজনে অতিরিক্ত প্রশ্ন করুন]

বাণিজ্যিক কিংবা ঘরে বানানো স্যালাইনের মাঝে কোনটা বেশি কার্যকরী?

দুটোর খরচ ও বানানোর প্রক্রিয়ার ব্যাপারে মতামত:

Q9. Please tell us about the use of tasty saline or fruit juice in diarrhoea.

প্রশ্ন ৯. পাতলা পায়খানাতে টেস্টি স্যালাইন কিংবা ফলের রসের ব্যাবহার সম্পর্কে আমাদের কিছু বলুন।

[Please probe where necessary]

[প্রয়োজনে অতিরিক্ত প্রশ্ন করুন]

টেস্টি স্যালাইনের ব্যাপারে মতামত:

বাণিজ্যিক ফলের রসের ব্যাপারে মতামত:

বাণিজ্যিক ফলের রস কিংবা অন্য কিছু খাওয়ালে তা স্যালাইনের ওপর প্রভাব ফেলে কি না:

চিনি কিংবা মিষ্টি জাতীয় রস যোগ করলে তা বাচ্চারা বেশি গ্রহণ করে কি না:

স্যালাইনের সঙ্গে পানি দেওয়া

অনান্য:

Q10. Please tell us about the side effects of using ORS, if any.

প্রশ্ন ১০: খাবার স্যালাইন ব্যবহারের কোনো পার্শ্বপ্রতিক্রিয়া থাকলে সে সম্পর্কে বলুন।

জবাব:

স্যালাইন খেলে বাচ্চা বমি করে প্রসঙ্গে:

অতিরিক্ত স্যালাইন প্রদানের পার্শ্বপ্রতিক্রিয়া:

সঠিকভাবে না বানাবার ফলাফল:

অন্যান্য:

Q11. Is there any merit in drinking ORS by a mother if her child is suffering from diarrhoea? Please elaborate.

প্রশ্ন ১১: বাচ্চার ডায়রিয়ায় মায়ের স্যালাইন খাওয়ার কোন উপকারিতা আছে বলে মনে করেন কি? দয়া করে বিস্তারিত বলুন।

[Please probe where necessary]

[প্রয়োজনে অতিরিক্ত প্রশ্ন করুন]

বাচ্চা বুকের দুধ খেলে, মায়ের স্যালাইন পানের উপকারিতা:

Q12. How, when and why do you prepare ORS?

প্রশ্ন ১২: আপনি নিজে কখন স্যালাইন প্রস্তুত করেন? দয়া করে বিস্তারিত বলুন।

[Please probe where necessary]

[প্রয়োজনে অতিরিক্ত প্রশ্ন করুন]

Q13. When would you need to prepare saline? Please explain in details

প্রশ্ন ১৩: আপনি নিজে কেন স্যালাইন প্রস্তুত করেন? দয়া করে বিস্তারিত বলুন।

[Please probe where necessary]

[প্রয়োজনে অতিরিক্ত প্রশ্ন করুন]

Q14. How do you yourself prepare ORS? Please explain in detail.

প্রশ্ন ১৪: আপনি নিজে কীভাবে স্যালাইন প্রস্তুত করেন? দয়া করে বিস্তারিত বলুন।

[Please probe where necessary]

[প্রয়োজনে অতিরিক্ত প্রশ্ন করুন]

প্রতিবার স্যালাইন বানানোর পূর্বে করণীয় (হাত ধোয়া, পাত্র পরিষ্কার করা):

স্যালাইন বানানোর সময় করণীয় (পরিমান মতো উপকরণ নেওয়া ও সঠিকভাবে মেশানো):

স্যালাইন বানাবার পর করণীয় (বাচ্চাকে কখন, কতটুকু কীভাবে খাওয়ানো):

প্রত্যেক মায়ের স্যালাইন তৈরির পদ্ধতি শেখা প্রসঙ্গে:

প্রত্যেক মায়ের স্যালাইন প্রদানের নিয়ম জানা প্রসঙ্গে:

ঘরে স্যালাইন বানানো প্রসঙ্গে:

ঘরে স্যালাইনের প্যাকেট রাখা প্রসঙ্গে:

স্যালাইনের পাশাপাশি পানি-জাতীয় খাবার প্রদান প্রসঙ্গে:

স্যালাইন ও অ্যান্টিবায়োটিকের আপেক্ষিক উপকারীতা প্রসঙ্গে:

Q15. When do you preserve saline? Please explain in detail.

প্রশ্ন ১৫: আপনি নিজে কখন স্যালাইন সংরক্ষণ করেন? দয়া করে বিস্তারিত বলুন।

[Please probe where necessary]

[প্রয়োজনে অতিরিক্ত প্রশ্ন করুন]

Ques 16: How do you preserve saline? Please explain in detail.

প্রশ্ন ১৬: আপনি নিজে কীভাবে স্যালাইন সংরক্ষণ করেন? দয়া করে বিস্তারিত বলুন।

[Please probe where necessary]

[প্রয়োজনে অতিরিক্ত প্রশ্ন করুন]

Q17. Why do you preserve saline? Please explain in detail

প্রশ্ন ১৭: আপনি নিজে কেন স্যালাইন সংরক্ষণ করেন? দয়া করে বিস্তারিত বলুন।

[Please probe where necessary]

[প্রয়োজনে অতিরিক্ত প্রশ্ন করুন]

Q18. Do you have any other comments regarding ORS preparation and use?

প্রশ্ন ১৮: স্যালাইন তৈরি এবং ব্যবহার সম্পর্কে আরও কিছু বলতে চাইলে বলুন?

জবাব:
